# Assisted reproduction and perinatal emotional wellbeing: findings from a longitudinal study

**DOI:** 10.1017/S0033291724002423

**Published:** 2024-12

**Authors:** Megan Galbally, Irene Bobevski, Karen Wynter, Beverley Vollenhoven

**Affiliations:** 1 School of Clinical Sciences, Monash University, Australia; 2 Mental Health Program, Monash Health, Australia; 3 Health Futures Institute, Murdoch University, Australia; 4 School of Nursing and Midwifery, Deakin University, Australia; 5 Department of Obstetrics and Gynaecology, Monash University, Australia; 6 Women's and Children's Program, Monash Health, Australia; 7 Monash IVF, Australia

**Keywords:** anxiety, assisted reproduction, attachment, parenting, perinatal depression

## Abstract

**Background:**

There have been inconsistent findings for an association between assisted reproductive technology (ART) and poorer perinatal emotional wellbeing. This study is to explore whether ART is associated with increased depression and depressive symptoms, anxiety symptoms and parenting stress, and poorer antenatal attachment, over the perinatal period from pregnancy to 12 months postpartum.

**Methods:**

This study drew on data collected within an ongoing cohort from 806 women including 42 who had conceived using ART, and all recruited in early pregnancy and followed to 12 months postpartum. Measures included the Structured Clinical Interview for the DSM, Edinburgh Postnatal Depression Scale, State and Trait Anxiety Inventory, Maternal Antenatal Attachment Scale and Parenting Stress Index.

**Results:**

Women who conceived with ART were no more likely to be depressed. They had lower depressive and anxiety symptoms in early pregnancy, higher antenatal attachment and lower parenting stress. However, women who conceived with ART had a significant increase in depressive and anxiety symptoms in late pregnancy which reduced in the postpartum and showed a distinct pattern compared to those who conceived naturally.

**Conclusions:**

This study found that women who conceived with ART did not have poorer emotional wellbeing across the perinatal period. However, in late pregnancy depressive and anxiety symptoms did rise and consideration of this clinically and in future research is warranted.

## Background

In 2019, 4.9% of all women who gave birth in Australia used some form of assisted reproduction technology (ART) (AIHW, 2021). Despite this, there remains a lack of clarity as to the relationship between the use of ART and depression (Chen, Wang, Zhang, Zhao, & Chen, 2019). This includes recent systematic reviews which have reported conflicting findings as to whether ART is associated with an increase in the risk of poorer perinatal emotional wellbeing including perinatal depressive symptoms, anxiety, parenting stress and poorer maternal fetal attachment, reflecting in part gaps in methodology and measurement (Chen et al., 2019; Ranjbar, Warmelink, & Gharacheh, 2020). The construct of perinatal emotional wellbeing that includes measures across depression, anxiety, antenatal attachment, and postpartum parenting stress is increasingly expected in perinatal mental health research, as each of these components of emotional wellbeing is closely inter-related: high levels of anxiety symptoms are frequently associated with perinatal depression and both anxiety and depression can influence antenatal attachment and postpartum parenting; as a result, distinguishing between these can be arbitrary (Aran, Lewis, Watson, & Galbally, 2022; Condon & Corkindale, 1997; Falah-Hassani, Shiri, & Dennis, 2017; Galbally & Lewis, 2017; Galbally, Watson, Boyce, Nguyen, & Lewis, 2020). Also increasingly recognized is the importance of understanding changes over this transition and the journey of parenthood across pregnancy and the first year postpartum.

There are several forms of ART, and two common forms are *in vitro* fertilization (IVF) and gamete intrafallopian transfer (GIFT). IVF includes ovarian stimulation followed by surgical removal of eggs from the ovary, then fertilization with sperm and returning the embryo(s) to the female reproductive tract five days later. GIFT consists of the collection of eggs directly from the ovaries, which together with sperm are injected into the fallopian tubes via laparoscopy immediately after egg collection. Undertaking ART can be stressful for couples, including financially, the time involved in undertaking ART, the physical impacts of interventions required, and the distress if multiple attempts are required (Massarotti et al., 2019). This has the potential for conception through ART to increase a woman's vulnerability to symptoms of poor mental health, including depression, anxiety, and stress. Poorer mental health may also negatively impact on prenatal attachment and lead to higher postpartum parenting stress (Condon & Corkindale, 1997; Moe, von Soest, Fredriksen, Olafsen, & Smith, 2018). While a pregnancy may alleviate the stress associated with the uncertainty of infertility treatment, it may also bring anxiety about ongoing maternal and fetal health in pregnancy and the pregnancy outcome (Negris et al., 2021).

Despite these potential impacts on mental health, a systematic review of psychological adjustment and assisted reproduction found in most studies, although not universally, there was no reported difference in depressive symptoms, and higher levels of maternal fetal attachment among women who had conceived with ART compared to those who conceived naturally (Gourounti, 2016). Out of the 20 studies included, none had utilized a diagnostic measure of mental health and as such were unable to report on depression, only depressive symptoms (Gourounti, 2016). A meta-analysis specifically examining the association between postpartum depressive symptoms and ART identified 14 studies; most studies assessed depressive symptoms at a single timepoint and relied solely on screening measures for depression, such as the Edinburgh Postnatal Depression Scale (EPDS) (Gressier et al., 2015). Overall, this meta-analysis did not find an association between depressive symptoms and ART, but did recommend that future studies measure both depression and depressive symptoms and at more than one timepoint, as well as include other covariates such as anxiety symptoms, maternal age, and socio-demographic factors which were frequently missing from the identified studies. In the most recent systematic review and meta-analysis published in 2019 examining perinatal depressive symptoms and ART, the authors identified 22 studies and concluded there was no association with depressive symptoms across most studies, although some variation across studies was found and again the authors commented on the paucity of studies that included diagnostic measures for depression and the reliance on screening measures, frequently administered only once in the included studies (Chen et al., 2019). A recent study examining data from the Finnish registry found the rate of psychotropic medication prescription was higher for those who were unsuccessful in fertility treatment than those who conceived using ART or those who conceived naturally; these findings suggest it may be ongoing infertility that impacts most on mental health (Goisis et al., 2023). None of these reviews or studies also examined other aspects of perinatal emotional wellbeing.

A recent literature review on ART and prenatal attachment identified 17 studies and overall found no difference in attachment in pregnancy in most studies (Ranjbar et al., 2020). The studies identified ranged from sample sizes of 25 to 297; four also measured depressive symptoms using the EPDS. None included studies that followed up parenting into the postpartum. In a study not included and utilizing data collected as part of the UK Millennium Cohort Study, no difference was identified in the quality of the parent–child relationship between those who had any form of ART and those who did not; this sample was followed until 14 years of age (Goisis & Palma, 2021). However, a gap remains with studies that examine both antenatal and postnatal parenting including attachment.

To address the identified gaps in current research this study aims to examine the relationship between ART and perinatal emotional wellbeing through including a diagnostic measure of depression assessing clinical depression in early pregnancy, as well as repeat assessments of depressive symptoms in early and late pregnancy and two time points in the first postpartum year. First, this study will aim to examine (a) the relationship between ART and clinical depression as well as depressive symptoms across pregnancy and the postpartum. The further aims are to examine the relationship of ART to other aspects of perinatal emotional wellbeing including (b) anxiety symptoms across pregnancy and the postpartum, (c) antenatal attachment in early and late pregnancy, and (d) postpartum parenting stress across the first year.

## Methods

### Sample

The Mercy Pregnancy Emotional Wellbeing Study (MPEWS) based in Victoria and Western Australia is a pregnancy cohort study (Galbally et al., 2017). Women were eligible to participate if they were less than 20 weeks’ pregnant. Exclusion criteria for the data in this paper included: bipolar or psychotic disorders, substance abuse disorder, child protection involvement, intellectual disability, serious pre-existing physical illness, and psychiatric illness requiring current acute inpatient admission.

For this study, the sample drawn from MPEWS consisted of 806 women including 42 who had assisted reproduction to conceive the current pregnancy with either IVF or GIFT. Women in the control group had all conceived naturally. Women provided informed, written consent before participating in the study and ethical review and approval was provided by the Mercy Health Human Research Ethics Committee and the Western Australia Health South Metropolitan Human Research Ethics Committee.

### Measures

#### Assisted reproduction (ART)

The use of assisted reproduction was included in the self-report questionnaire administered at recruitment. This included items asking if the participant required medical help to conceive this baby and if so, further questions followed on the use of donor eggs, sperm, or embryos; whether stimulation of follicle development took place (with three options); whether assistance with fertilization was utilized (with five options), and whether frozen embryos were used. For this study we utilize data from women who responded that fertilization was by IVF (*in vitro* fertilization) or GIFT (sperm and eggs placed in fallopian tube); women who had used other forms of ART were not included in this nested study.

#### Maternal depression and anxiety

At recruitment at less than 20 weeks of pregnancy, the Structured Clinical Interview for DSM-IV (SCID-IV) was administered to identify current depressive disorders (First, Gibbon, Spitzer, Williams, & Benjamin, 1997). Depressive symptoms were measured in early pregnancy, late pregnancy, six, and 12 months postpartum using the EPDS (Cox, Holden, & Sagovsky, 1987), which has been validated for use with Australian women during the perinatal period (Boyce, Stubbs, & Todd, 1993). The State and Trait Anxiety Inventory (Spielberger, Gorsuch, Lushene, Vagg, & Jacobs, 1983) State Anxiety subscale (STAIS) was used to measure self-reported symptoms of state anxiety over the same four time points in the perinatal period.

#### Maternal antenatal attachment

The Maternal Antenatal Attachment Scale (MAAS) was administered to participants in early and late pregnancy. This scale is a 19-item self-report measure asking the respondent about their emerging feelings and cognitions about their unborn baby (Condon & Corkindale, 1997). The MAAS consists of two subscales: Quality and Intensity of maternal attachment. The MAAS can also be used as a total score: MAAS Global. The MAAS has previously been shown to have good construct validity and internal consistency (Van den Bergh & Simons, 2009).

#### Parenting stress

At six and 12 months postpartum, stress due to the roles of parenting was assessed using the fourth edition Parenting Stress Index (PSI), Short-form (Abidin & Psychological Assessment Resources, 2012). The 36-item PSI-4-SF divides into three subscales (Difficult Child, Parent Distress, and Parent–child Dysfunctional Interactions) and provides percentiles for comparison across studies. Due to strong concurrent associations between the Parent Distress subscale and the EPDS, we used only the Difficult Child (PSI DC) and Parent–Child Dysfunctional Interactions (PSI PCDI) subscale percentile scores.

### Statistical analysis

Sociodemographic and other descriptive characteristics were compared for statistically significant differences between the IVF/GIFT and the natural conception/ no ART intervention group using χ^2^ for frequencies or *t* tests for mean scores. For comparisons of frequencies with expected counts <5, Fisher's exact test was used. Significance testing for differences between birth outcomes, such as the infant physiological measurements, was adjusted for relevant pregnancy variables (see Table 2).

Mixed effects linear models were used to analyze how the trajectory of each mental health (EPDS and STAI), maternal attachment (MAAS Quality and MAAS Intensity), and parenting stress (PSI DC and PSI PCDI) outcome differed by IVF/GIFT status, after controlling for diagnosed depressive disorder at recruitment and relevant covariates. Time was modelled continuously. Quadratic time effects (Time^2^) were tested for the outcomes with more than two time points (EPDS and STAIS). For the outcomes with more than two time points, random effects for intercept and slope were estimated, as well as correlated using an unstructured error covariance assumption. For outcomes with only two time points (MAAS and PSI), a random effect for intercept was estimated.

For each outcome an unadjusted model was tested first, which included interaction of IVF/GIFT status with time and with the quadratic term of time (Time^2^). An adjusted model, controlling for depressive disorder at recruitment and relevant covariates, was then tested. Sociodemographic characteristics which were significantly different between the two groups were used as covariates in the analysis models. Tertiary education was also included as a covariate as an indication of socioeconomic status, since the literature indicates women conceiving through IVF/GIFT have higher socioeconomic status (Fisher et al., 2013). The interaction between IVF/GIFT status and depressive disorder was also examined. Where a significant interaction existed, differences of the estimated means between the ART/GIFT and the non-intervention group were compared at each time point using the 95% confidence interval (CI) of the mean difference for each comparison.

The analysis was conducted with Stata 18 (StataCorp, 2023).

## Results

### Demographics and descriptive characteristics

The demographic, pregnancy, and birth characteristics of the participants by IVF/GIFT status are presented in Tables 1 and 2. The majority of participants were partnered, tertiary educated, of Oceanic/European background and nulliparous at recruitment. Women who had received IVF/GIFT interventions were significantly older, more likely to have had delivery by elective Caesarean and less likely to have had unassisted vaginal delivery. There were no other significant differences in demographic, pregnancy, or birth characteristics between the two groups.

**Table 1.** Demographics and descriptive characteristics (unadjusted data)

	IVF/GIFT *n* = 42	Natural conception *n* = 764	*p*
Sociodemographic characteristics			
	*n* (%)	*n* (%)	
Not currently in a relationship (missing = 37)	3/39 (7.7)	16/730 (2.2)	0.067^†^
Tertiary education (missing = 3)	31/42 (73.8)	457/761 (60.1)	0.106
Oceanic/European background (missing = 0)	38/42 (90.5)	669/764 (87.6)	0.750
Nulliparous (missing = 3)	33/42 (78.6)	506/761 (66.5)	0.146
Mental health			
Depressive disorder at recruitment (missing = 0)	6/42 (14.3)	145/764 (19.0)	0.578
	M (s.d., n)	M (s.d., n)	
Maternal age at recruitment (missing = 0)	36.4 (5.3, 42)	31.5 (4.6, 764)	<0.001
Birth and pregnancy			
	*n* (%)	*n* (%)	
Mode of delivery (missing = 39)			<0.001^†^
Vaginal non-assisted	8/41 (19.5)^a^	339/726 (46.7)^b^	
Vaginal assisted	11/41 (26.8)	152/726 (20.9)	
Emergency Caesarean	11/41 (26.8)	171/726 (23.6)	
Elective Caesarean	11/41 (26.8)^a^	64/726 (8.8)^b^	
BMI at recruitment ⩾30 (missing = 5)	12/42 (28.6)	172/759 (22.7)	0.381
Smoking during pregnancy (missing = 46)	1/40 (2.5)	65/720 (9.0)	0.244^†^
Alcohol consumption during pregnancy (missing = 46)	7/40 (17.5)	178/720 (24.7)	0.300
Hypertensive disorder of pregnancy (missing = 51)	4/39 (10.3)	41/716 (5.7)	0.282^†^
Pregnancy-induced hypertension (missing = 51)	1/39 (2.6)	26/716 (3.6)	0.999^†^
Gestational diabetes mellitus (missing = 45)	4/40 (10.0)	87/721 (12.1)	0.934^†^
Pre-eclampsia (missing = 51)	3/39 (7.7)	17/716 (2.4)	0.079^†^
Breast feeding (missing = 169)			0.606^†^
Never breastfed	2/37 (5.4)	20/600 (3.3)	
Ceased prior to 6 months	10/37 (27.0)	158/600 (26.3)	
Ceased prior to 12 months	25/37 (67.6)	422/600 (70.3)	
	M (s.d., n)	M (s.d., n)	
BMI at recruitment (missing = 5)	27.38 (5.3, 42)	26.67 (5.5, 759)	0.418
Infant gestational age at delivery (weeks) (missing = 52)	38.85 (1.7, 40)	39.22 (1.9, 714)	0.230

M, mean; SD, standard deviation.

*Note*: For significance testing, χ^2^ tests were used for frequencies and *t* tests for means. Valid percentages are reported, with missing data being handled casewise.

† Significance testing carried out with Fisher's exact test due to expected cell counts <5.

^a,b^, Differing superscript letters between cells denote sub-categories which are significantly different between women with IVF/GIFT and no medical intervention, as indicated by adjusted standardized residuals >2.

**Table 2.** Estimated marginal means or proportions for birth outcomes and breastfeeding

	IVF/GIFT M (95% CI)	Natural conception M (95% CI)	*p*
Standardised birth weight^a^	−0.07 (−0.39, 0.26)	−0.09 (−1.16, −0.02)	0.883
Birth length (cm)^b^	50.58 (49.88, 51.28)	50.38 (50.21, 50.55)	0.597
Birth head circumference^b^	34.60 (34.12, 35.05)	34.38 (34.27, 34.50)	0.374
APGAR at 1 min^c^	8.49 (8.11, 8.87)	8.13 (8.02, 8.24)	0.068
APGAR at 5 min^c^	9.05 (8.88, 9.21)	8.91 (8.85, 8.98)	0.153
Breast feeding^d^	% (95% CI)	% (95% CI)	
Percentage of women breast feeding up to 12 months	62.09 (43.94, 80.24)	72.00 (68.24, 75.77)	0.296

M, mean; CI, confidence interval.

a Model adjusted for maternal age and smoking at recruitment, and mean pregnancy EPDS score.

b Model adjusted for maternal age at recruitment, mean pregnancy EPDS score, and infant gender and gestational age at delivery.

c Model adjusted for maternal age at recruitment, mean pregnancy EPDS score, and infant gestational age at delivery.

d Model adjusted for maternal age, tertiary education, and mean postpartum EPDS score.

### Results from mixed models

**Figure 1.**
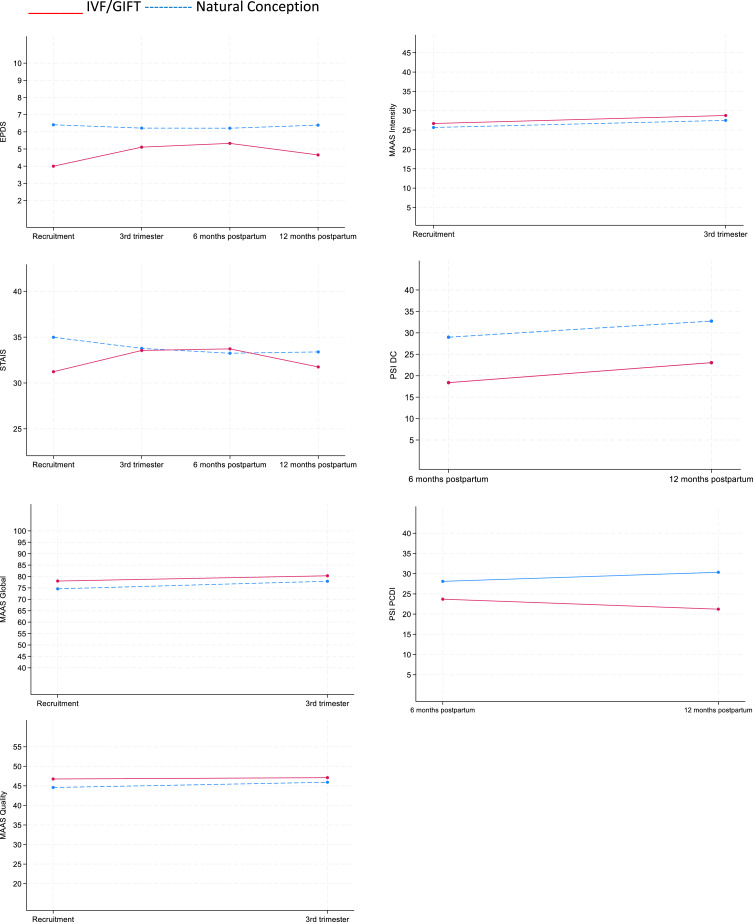
Plots of estimated marginal means derived from the adjusted mixed models.

The models’ estimates are shown in Table 3, and the marginal mean trajectories by IVF/GIFT status are plotted in Fig. 1. None of the covariates made a substantial difference to the trajectories examined by each model. In the final adjusted models in Table 3 only the statistically significant covariates were retained.

**Table 3.** Mixed effects regression models by IVF/GIFT status

	Unadjusted model			Adjusted model		
	*b*	95% CI	*p*	*b*	95% CI	*p*
**EPDS** (*n* = 805)						
Intercept	6.44	6.09, 6.79	⩽0.001	6.06	5.55, 6.56	<0.001*
Time	−0.29	−0.65, 0.06	0.103	−0.29	−0.64, 0.68	0.113
Time^2^	0.09	−0.02, 0.21	0.115	0.09	−0.02, 0.21	0.115
IVF/GIFT	−2.35	−3.88, −0.82	0.003*	−2.41	−3.88, −0.95	0.001*
Time*IVF/GIFT	1.87	0.36, 3.37	0.015*	1.84	0.34, 3.35	0.016*
Time^2^*IVF/GIFT	−0.54	−1.02, −0.06	0.028*	−0.54	−1.02, −0.06	0.029*
Depressive disorder	–	–	–	3.99	3.29, 4.68	<0.001*
Maternal age	–	–	–	0.07	0.01, 0.13	0.019*
Tertiary education	–	–	–	−0.59	−1.15, −0.02	0.042*
Random effects parameters	Estimate	95% CI		Estimate	95% CI	
Variance of the random intercept	16.67	14.54, 19.11		13.65	11.79, 15.80	
Variance of the random slope	0.94	0.66, 1.35		0.93	0.64, 1.34	
Covariance the random intercept and slope	−1.47	−2.18, −0.76		−1.27	−1.94, −0.60	
**STAI** (*n* = 802)						
Intercept	35.07	34.27, 35.86	⩽0.001	32.34	30.95, 33.73	<0.001*
Time	−1.52	−2.32, −0.71	⩽0.001*	−1.55	−2.36, −0.73	<0.001*
Time^2^	0.32	0.06, 0.59	0.018*	0.34	0.07, 0.61	0.014*
IVF/GIFT	−3.99	−7.48, −0.52	0.024*	−3.76	−7.08, −0.43	0.027*
Time*IVF/GIFT	4.78	1.34, 8.23	0.007*	4.94	1.46, 8.42	0.005*
Time^2^*IVF/GIFT	−1.34	−2.45, −0.23	0.018*	−1.41	−2.53, −0.29	0.014*
Depressive disorder	–	–	–	8.88	7.36, 10.40	<0.001*
Mode of birth						
Vaginal unassisted	–	–	–	1.28	−0.27, 2.82	0.106
Vaginal assisted	–	–	–	Reference	–	–
Emergency Caesarean	–	–	–	0.91	−0.85, 2.67	0.311
Elective Caesarean	–	–	–	2.59	0.31, 4.87	0.026*
Random effects parameters	Estimate	95% CI		Estimate	95% CI	
Variance of the random intercept	84.22	73.24, 96.83		62.34	53.07, 73.33	
Variance of the random slope	3.18	1.94, 5.23		2.69	1.49, 4.85	
Covariance the random intercept and slope	−8.34	−11.87, −4.80		−5.79	−9.08, −2.50	
**MAAS Global** (*n* = 806)						
Intercept	74.67	74.13, 75.22	⩽0.001*	75.01	74.42, 75.59	<0.001*
Time	3.31	2.91, 3.72	⩽0.001*	3.32	2.91, 3.72	<0.001*
IVF/GIFT	2.26	−0.13, 4.65	0.064	3.42	2.91, 3.72	0.005*
Time*IVF/GIFT	−0.99	−2.72, 0.74	0.260	−1.02	−2.75, 0.71	0.246
Depressive disorder	–	–	–	−2.12	−3.39, −0.86	0.001*
Maternal age	–	–	–	−0.26	−0.37, −0.16	<0.001*
Random effects parameters	Estimate	95% CI		Estimate	95% CI	
Variance of the random intercept	44.37	39.47, 49.88		42.45	37.72, 47.77	
**MAAS Quality** (*n* = 806)						
Intercept	44.59	44.33, 44.86	⩽0.001*	45.23	44.86, 45.61	<0.001*
Time	1.33	1.11, 1.55	⩽0.001*	1.34	1.12, 1.56	<0.001*
IVF/GIFT	1.81	0.65, 2.98	0.002*	2.17	1.01, 3.34	<0.001*
Time*IVF/GIFT	−0.99	−1.93, −0.05	0.039*	−1.00	−1.95, −0.05	0.040*
Depressive disorder	–	–	–	−1.35	−1.95, −0.75	<0.001*
Maternal age	–	–	–	−0.07	−0.12, −0.02	0.009*
Mode of birth						
Vaginal unassisted	–	–	–	Reference		
Vaginal assisted	–	–	–	−0.74	−1.35, −0.13	0.018*
Emergency Caesarean	–	–	–	−0.74	−1.33, −0.15	0.014*
Elective Caesarean	–	–	–	−0.55	−1.38, 0.29	0.198
Random effects parameters	Estimate	95% CI		Estimate	95% CI	
Variance of the random intercept	9.61	8.48, 10.89		8.46	7.42, 9.64	
**MAAS Intensity** (*n* = 806)						
Intercept	25.72	25.39, 26.05	⩽0.001*	25.81	25.45, 26.17	<0.001*
Time	1.83	1.57, 2.09	⩽0.001*	1.83	1.57, 2.09	<0.001*
IVF/GIFT	0.25	−1.20, 1.71	0.733	1.03	−0.44, 2.49	0.171
Time*IVF/GIFT	0.26	−0.85, 1.37	0.646	0.24	−0.87, 1.34	0.674
Depressive disorder	–	–	–	−0.69	−1.45, 0.08	0.077
Maternal age	–	–	–	−0.17	−0.23, −0.10	<0.001*
Random effects parameters	Estimate	95% CI		Estimate	95% CI	
Variance of the random intercept	15.89	14.09, 17.91		15.28	13.54, 17.25	
**PSI DC** (*n* = 629)						
Intercept	29.21	27.25, 31.16	⩽0.001	23.87	19.94, 27.80	<0.001*
Time	3.61	1.49, 5.73	0.001*	3.75	1.62, 5.89	0.001*
IVF/GIFT	−10.77	−19.44, −2.10	0.015*	−10.60	−19.28, −1.92	0.009*
Time*IVF/GIFT	1.63	−7.00, 10.26	0.711	0.91	−7.72, 9.54	0.837
Depressive disorder	–	–	–	9.78	5.33, 14.22	<0.001*
Mode of birth						
Vaginal unassisted	–	–	–	4.82	0.31, 9.32	0.036*
Vaginal assisted	–	–	–	Reference	–	–
Emergency Caesarean	–	–	–	3.46	−1.63, 8.55	0.182
Elective Caesarean	–	–	–	4.07	−2.60, 10.74	0.232
Random effects parameters	Estimate	95% CI		Estimate	95% CI	
Variance of the random intercept	321.51	269.10, 384.13		308.26	256.80, 370.04	
**PSI PCDI** (*n* = 628)						
Intercept	28.22	26.48, 29.97	⩽0.001	26.56	24.68, 24.42	<0.001*
Time	2.12	0.26, 3.98	0.026*	2.24	0.38, 4.11	0.018*
IVF/GIFT	−4.68	−12.39, 3.98	0.234	−4.41	−12.02, 3.20	0.256
Time*IVF/GIFT	−4.78	−12.33, 2.78	0.216	−4.72	−12.27, 2.83	0.220
Depressive disorder	–	–	–	8.92	4.96, 12.88	<0.001*
Random effects parameters	Estimate	95% CI		Estimate	95% CI	
Variance of the random intercept	265.43	233.65, 315.02		254.52	213.87, 302.91	

*b*, regression coefficient; CI, confidence interval; EPDS, Edinburgh Postnatal Depression Scale; STAI, State and Trait Anxiety Inventory; MAAS, Maternal Antenatal Attachment Scale; PSI, Parenting Stress Scale; DC, Difficult Child Subscale; PCDI, Parent Child Dysfunctional Interactions Subscale; Reference, Reference category.

* p < 0.05.

### Depressive symptoms and assisted reproduction

A non-linear (quadratic) interaction was found between IVF/GIFT status and time. Women with IVF/GIFT had a significantly lower level of depressive symptoms in early pregnancy (mean difference = −2.41, 95% CI −3.87 to −0.95). However, during early and late pregnancy there was no significant difference between the two groups. Then at 6 and 12 months postpartum, the symptoms of the IVF/GIFT group were again slightly, but significantly, lower compared to the non-intervention group (mean difference = −1.73, 95% CI −3.35 to −0.11).

### Anxiety and assisted reproduction

A non-linear (quadratic) interaction was found between IVF/GIFT status and time. The IVF/GIFT group had a significantly lower level of anxiety symptoms in early pregnancy (mean difference = −3.76, 95% CI −7.08 to −0.43), but not from late pregnancy to 12 months postpartum.

### Maternal attachment and assisted reproduction

There were no significant interactions between IVF/GIFT status and time for the total MAAS Global scale, and the MAAS Intensity subscale. The effect of IVF/GIFT did not reach significance for the MAAS Intensity subscale. For the MAAS Quality subscale, a significant interaction was found between IVF/GIFT status and time. At recruitment women with an IVF/GIFT intervention scored significantly higher on attachment quality than those without such an intervention (mean difference = 2.17, 95% CI 1.01–3.34). However, at the third trimester there was no significant difference between the two groups.

### Parenting stress and assisted reproduction

For the PSI DC subscale, there was no significant interaction between IVF/GIFT status and time. However, IVF/GIFT contributed significantly to lower parental stress levels on average, compared to the non-intervention group.

For the PSI PCDI subscale, there was also no significant interaction between IVF/GIFT status and time. The effect of IVF/GIFT did not reach significance.

### Interpretation of random effects parameters

Table 3 shows that the random intercepts of all of the above analyses had significant variances, as indicated by their confidence intervals. This means that the intercepts (i.e. the baseline scores at recruitment), varied significantly between individuals for each measure. For the EPDS and the STAIS the variances of the random slopes were also significant. This indicates a significant variation in the slope (i.e. rate of change over time) in depressive and anxiety symptoms between individuals. The significant negative covariances between the random slope and intercept of the EPDS and STAIS indicate that for individuals with higher baseline scores (intercepts) there tends to be a lower rate of change over time and *vice versa*. The use of mixed models with random intercepts and slopes is thus appropriate for this data, as it accounts for individual variations.

### Interaction between IVF/GIFT and depressive disorder

There was no significant interaction between IVF/GIFT and depressive disorder, and no three-way interaction among IVF/GIFT, depressive disorder, and time in any of the models. However, depressive disorder at less than 20 weeks gestation significantly contributed to higher levels of depressive and anxiety symptoms, reduced maternal attachment, and higher parenting stress.

## Discussion

Our study examined the relationship between ART and perinatal emotional wellbeing including depression, depressive symptoms, anxiety symptoms, antenatal attachment, and postpartum parenting stress over pregnancy and the postpartum (Galbally, Blankley, Power, & Snellen, 2013). This study did not find an association between ART and depression or perinatal depressive symptoms and adds to previous findings through the inclusion of a diagnostic measure of clinical depression early in pregnancy and repeat measurement of depressive symptoms across pregnancy and the postpartum (Chen et al., 2019). Women who had conceived with ART, while being no more likely to be depressed, scored significantly lower on depressive symptoms at recruitment, reported a similar level of depressive symptoms to that of women without ART in late pregnancy and early postpartum, and then again reported a lower level of symptoms at 12 months postpartum. This pattern did differ to women who conceived naturally. A similar pattern for anxiety symptoms in women who conceived with ART was found, with women who had conceived through IVF/GIFT scoring lower on anxiety symptoms at recruitment, but then reporting the same level as women who did not conceive with IVF/GIFT. The quality of maternal antenatal attachment in women who conceived with ART commenced at higher levels in early pregnancy, but in late pregnancy the difference to those who conceived naturally was no longer significant. For parenting stress, women who conceived with ART had lower parenting stress at 6 and 12 months on the Difficult Child Subscale, but for both groups of women, parenting stress was noted to increase over the first year.

This study unsurprisingly found that women who had conceived using ART were more likely to be older and were more likely to then have a caesarean delivery than women who conceived naturally, consistent with other studies (Lodge-Tulloch et al., 2021). Within our sample there was no difference between women who conceived using IVF or GIFT and those who conceived naturally in demographic factors other than maternal age, pregnancy complications, birth outcomes, or breastfeeding rates.

There is no doubt that conceiving a child through ART can have impacts on families including financially, physically, and emotionally, and the journey can be unpredictable with the risk of loss through unsuccessful attempts or loss of pregnancies (Massarotti et al., 2019; René, Landry, & de Montigny, 2022). This study provides reassurance that despite these challenges women who conceive through ART have no greater risks of poorer perinatal emotional wellbeing including depression, anxiety, antenatal attachment, and parenting stress. This confirms previous studies and builds on these through the use of repeat and robust measures including a diagnostic measure for depression, a gap highlighted repeatedly in previous systematic reviews (Chen et al., 2019; Gressier et al., 2015; Ranjbar et al., 2020). It could be speculated that for the women included in this study the successful outcome of conceiving using ART underlies the findings of lower depressive and anxiety symptoms and higher attachment in pregnancy and that the rise in anxiety and depressive symptoms in late pregnancy may be associated with the transition from successful achievement of pregnancy to concerns about delivery and the birth of a healthy baby. The reduction in depressive symptoms over the postpartum would support this speculation; however additional data, including qualitative, would be required to confirm this. In a recent systematic review of qualitative studies examining the experience of conception using ART (René et al., 2022) the authors identified common themes including women feeling a sense of ‘fragility and vulnerability’ across pregnancy and perceiving the pregnancy as at risk of loss resulting in anxiety and emotional impacts including fear of losing the baby (René et al., 2022). While our study cannot attribute the rise in depressive and anxiety symptoms in late pregnancy to a specific cause, our findings do suggest that supporting the emotional wellbeing in later pregnancy for women who have conceived using ART is warranted.

While this study had many strengths, the limitations include the absence of data on partners’ and fathers’ emotional wellbeing as well as partner and family support given both these factors may be important in understanding a relationship among mental health, parenting, and reproductive assistance. This study also did not collect qualitative data on women's experience of reproductive assistance, pregnancy, and parenting; such individual experiences and perspectives would broaden the data and insight into understanding reproductive assistance and mental health. This includes understanding what may be underlying the rise in depressive and anxiety symptoms in late pregnancy noted in our findings. Owing to small numbers in individual categories, we were also unable to present data on wider ART beyond IVF and GIFT, for example oocyte donation or intracytoplasmic sperm injection, which may have different impacts on perinatal emotional wellbeing. A further limitation is as this sample was drawn from an existing cohort not designed to specifically recruit women who had undergone ART, these findings may not generalize to the range of groups of women who undertake ART; these include single women, as most women were partnered in this sample, and LGBTQIA+ people. Additional limitations include lack of inclusion of a history of depressive disorders prior to pregnancy and we did not assess for adjustment disorders. Although the random effect terms indicate significant individual variations in the slopes, due to the relatively small number of ART/GIFT participants, it is not feasible to explore further whether there are distinct groups of women with differing trajectories (such as different latent classes).

An increasing number of women and couples are undertaking ART to conceive pregnancy with potential financial, emotional, and physical impacts, as well as the hope this provides to overcome infertility and have a healthy baby. Our study found that those women who conceived using ART were no more likely to have poorer emotional wellbeing including depression, anxiety, antenatal attachment, and postpartum parenting stress than those who conceive naturally; this is a reassuring finding for women and their families. In fact, women who conceived using ART were found to have lower depressive and anxiety symptoms, higher antenatal attachment, and lower postpartum parenting stress than those who conceived naturally. A significant rise was noted in depressive and anxiety symptoms in late pregnancy for women who conceived with ART, and further research is needed to understand how to support women who have conceived with ART at this time in the perinatal period. Research replicating this and clarifying whether, as this coincided with late pregnancy, this rise in symptoms is associated with concerns about delivering a healthy baby, concerns about the risk of preterm birth, or other unknown concerns at this time would assist with developing and delivering effective support tailored to this group of women. The overall lower depressive and anxiety symptoms and depressive disorders in early pregnancy and higher levels of antenatal attachment in women who conceived with ART suggest despite the challenges of ART there is much resilience among women who conceive with ART.
